# Irradiation Induced Microstructure Evolution in Nanostructured Materials: A Review

**DOI:** 10.3390/ma9020105

**Published:** 2016-02-06

**Authors:** Wenbo Liu, Yanzhou Ji, Pengkang Tan, Hang Zang, Chaohui He, Di Yun, Chi Zhang, Zhigang Yang

**Affiliations:** 1 Department of Nuclear Science and Technology, Xi’an Jiaotong University, Xi’an 710049, China; tanpengkang@stu.xjtu.edu.cn (P.T.); zanghang@xjtu.edu.cn (H.Z.); hechaohui@xjtu.edu.cn (C.H.); diyun1979@xjtu.edu.cn (D.Y.); 2 Department of Materials Science and Engineering, The Pennsylvania State University, University Park, PA 16802, USA; yxj135@psu.edu; 3 Key Laboratory of Advanced Materials of Ministry of Education, School of Materials Science and Engineering, Tsinghua University, Beijing 100084, China; chizhang@tsinghua.edu.cn (C.Z.); zgyang@tsinghua.edu.cn (Z.Y.)

**Keywords:** nanostructured materials, irradiation resistance, grain boundary, void denuded zones

## Abstract

Nanostructured (NS) materials may have different irradiation resistance from their coarse-grained (CG) counterparts. In this review, we focus on the effect of grain boundaries (GBs)/interfaces on irradiation induced microstructure evolution and the irradiation tolerance of NS materials under irradiation. The features of void denuded zones (VDZs) and the unusual behavior of void formation near GBs/interfaces in metals due to the interactions between GBs/interfaces and irradiation-produced point defects are systematically reviewed. Some experimental results and calculation results show that NS materials have enhanced irradiation resistance, due to their extremely small grain sizes and large volume fractions of GBs/interfaces, which could absorb and annihilate the mobile defects produced during irradiation. However, there is also literature reporting reduced irradiation resistance or even amorphization of NS materials at a lower irradiation dose compared with their bulk counterparts, since the GBs are also characterized by excess energy (compared to that of single crystal materials) which could provide a shift in the total free energy that will lead to the amorphization process. The competition of these two effects leads to the different irradiation tolerance of NS materials. The irradiation-induced grain growth is dominated by irradiation temperature, dose, ion flux, character of GBs/interface and nanoprecipitates, although the decrease of grain sizes under irradiation is also observed in some experiments.

## 1. Introduction

Nanostructured (NS) materials are materials of which the microstructure has a characteristic length scale of just a few nanometers (1–2 to ~100 nm) [1,2]. According to the shape of the crystallites, there are three categories of NS materials: layer-shaped crystallites, rod-shaped crystallites, and NS materials composed of equiaxed nanometer-sized crystallites [1]. For all three categories of NS materials, the grain boundaries (GBs)/interfaces and triple junctions increase substantially with a decrease in the average grain size. For instance, the fraction of interfaces in the whole volume of the material can reach ~50% when the effective grain size is ~6 nm [3]. Hence, NS materials usually have different properties compared with their coarse-grained (CG) counterparts, due to their extremely small grain sizes and large volume fractions of GBs/interfaces.

The microstructure of materials has an important effect on the point defects produced by the displacement cascades during irradiation. The so-called displacement cascades during irradiation involve the Frenkel pairs in a form of the interstitial atoms and vacancies (IAV), as well as their clusters in a form of loops or the voids [4,5]. During a collision cascade, atoms are displaced from their lattice sites by the high-energy particle, and most of these atoms occupy new lattice sites, which can form displacement spike. The region of the displacement spike in a single crystal material is quite large due to the channeling effects, which is quite different from that in polycrystalline materials, since GBs/interfaces in polycrystalline materials can absorb the irradiation-induced point defects and their clusters.

Since GBs/interfaces are known to act as sinks for defects of all types, it is expected that NS materials are likely to have different radiation damage tolerance compared with their CG materials counterparts. In the past several decades, much attention was paid to the investigation of the NS materials under various irradiation conditions, and most of the previous reviews were focused on the irradiation resistance of the NS materials composed of equiaxed nanometer-sized crystallites [3,4,6] or layer-shaped crystallites (multilayer materials) [7] under irradiation. However, understanding of the irradiation tolerance of all NS materials, from the viewpoint of the role and effect of the GBs/interfaces in materials, is still needed.

Although different particle types (*i.e.*, electron, proton, neutron, heavy ion, *etc.*) have different advantages and disadvantages in the study of irradiation effects [5], the summary of their advantages and disadvantages is beyond the scope of the present review. Nevertheless, the type of the particle is clearly given in each of the involved experiments in the present review. In the present review, effect of GBs/interfaces on microstructure evolution during irradiation and irradiation induced microstructure evolution in NS materials are systematically reviewed. Effect of GBs/interfaces on microstructure evolution during irradiation is presented in Section 2. The irradiation induced microstructure evolution of NS materials, concerning enhanced irradiation resistance, irradiation-induced amorphization and irradiation-induced grain growth are presented in Section 3.

## 2. Effect of GBs/Interfaces on Irradiation-Induced Microstructure Evolution

In the past several decades, voids are frequently observed in metals by neutron irradiation [8], ion irradiation [9] and electron irradiation [10]. Since GBs/interfaces are usually regarded as effective sinks of point defects produced during irradiation, it is expected that void-denuded zones (VDZs) can be observed near the GBs/interfaces due to its absorption of vacancies in GBs. In fact, denudation of voids along GBs and around twin bands were already observed in ion-irradiated nickel [11,12], copper [13,14] and stainless steel [15,16,17], and the denuded zone has an important effect on the swelling behavior of materials during low energy ion irradiation [12]. The width of the VDZs is usually taken as the distance between the GBs/interfaces and the area where the void density is half of its normal value within the grain [18].

**Figure 1:**
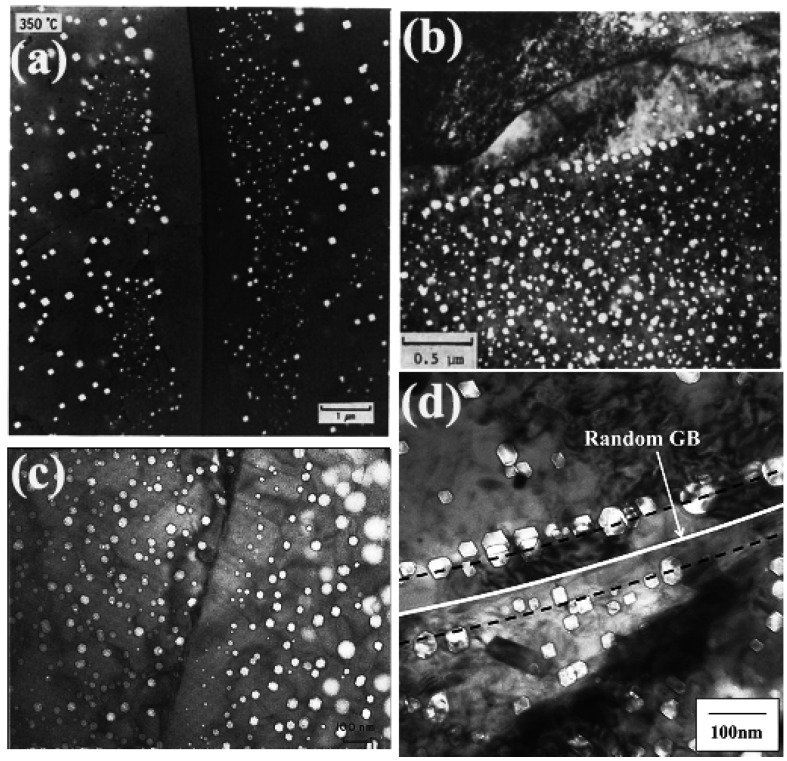
Morphology of void denuded zones (VDZs) along grain boundaries (GBs) in (**a**) copper irradiated at 330 °C [14]; (**b**) 304 stainless steel irradiated at 625 °C [17]; (**c**) nickel irradiated at high temperature [12]; and (**d**) PNC316CW stainless steel neutron-irradiated at 775 K to 103 dpa [16].

As shown in Figure 1, VDZs have been observed in many irradiated materials. There are three salient features of the VDZs along GBs [11], which can be described as follows:

(1) Many VDZs only appear on one side of the GBs, while no denuded zones appear on the other side. The uneven denuded zone was first reported by Brimhall and Mastel [19], and they pointed out that the zone is much wider on one side of the boundary than on the other. By tilted transmission electron microscopy (TEM) foils, it is concluded that this one-sided denudation was not caused by improper positioning of the TEM foils, but reflects the physical conditions of the GBs during the ion irradiation [11]. In fact, the one-side appearance of VDZs at many of the GBs is caused by the migration of these boundaries. The VDZs are originally induced symmetrically with respect to the center of the GBs, but the GBs are forced to migrate in order to decrease the internal stresses acting on them. The movement of GBs would encounter little resistance in the VDZs due to the lack of voids as well as other point defects in the region. Hence, the moving GBs can easily pass through the VDZs until obstacles such as voids or other defects are present. This can be used to explain why the VDZs on the one side of GBs are wider than on the other side. Moreover, ion irradiation induced GBs migration has been observed both in experiments [12,20] and calculations [21,22], which can be used to support this explanation.

(2) The VDZs along GBs usually exhibit an enhancement of void formation at the edges of the zone, and unusually large voids are formed in one or two rows in the edge. This phenomenon seems to be closely related to the first feature. But the formation of the unusually large voids at the edge of the VDZs may be detrimental particularly not only in swelling, but also in the mechanical property when the voids there begin to interconnect. It is generally accepted that the formation of this void “wall” or voids of larger sizes at the edges of the VDZs is due to the difference between the mobility of vacancies and interstitials. The interstitials could be attracted and adsorbed by the GBs from a wider band on both sides of the GBs than the band for vacancies, and the overlap of these two bands leads to the appearance of the VDZs. However, the existence of the region, where excess vacancies are created on each side of the GBs, leads to the rapid growth of voids. This theory can be used to explain the formation of the unusually large voids at the edge of the VDZs, but it could not explain the observed dependence of the large void sizes upon the width the one-sided denuded zone [11]. Another alternative explanation of the unusually large voids formed in the edge of the VDZs is the interaction of dislocations with the GBs, and the existence of a band with a different density of dislocations relative to the bulk during irradiation [9]. If this band is wider than the VDZs, one could expect different void formation characteristics. Moreover, the dislocations also more readily absorb interstitials than vacancies [23,24].

(3) “Giant” voids appear occasionally inside the VDZs. Voids with sizes larger than 100 nm are observed, though infrequently, in the middle of the denuded zone. Due to the association with other impurity atoms, some free bubbles could migrate sluggishly or would be anchored inside the VDZs. There would also be the possibility that bubbles could have been formed accidentally in the VDZs prior to the movement of the boundary. The growth of the preexisting bubbles leads to the formation of the “giant” voids in the middle of the VDZs, since they are the only sinks for vacancies in the whole denuded zone [11]. Hence “giant” voids occasionally emerge inside the VDZs.

The irradiation conditions, such as irradiation dose, dose rate and temperature, can significantly affect the formation of VDZs near GBs [14,25,26,27]. It is reported that the width of VDZs decreases with increasing dose and increases with increasing temperature [12], and a critical irradiation dose is reported to be crucial for a clear VDZ [28]. Due to the significantly low dose rate (2 × 10^−7^ dpa·s^−1^) [14,25], the neutron radiation-induced defects have much more time to migrate to GBs, leading to the formation of VDZs. As suggested by Zinkle [29], if there is a critical vacancy concentration that is needed for voids to nucleate and grow, the VDZs could form near GBs. The effect of temperature on the point defect migration and precipitation in materials is remarkable [30], and thus the temperature has an important effect on the irradiation induced microstructure changes. For lower irradiation temperature, the vacancy diffusivity is relative lower, leading to higher vacancy supersaturations and therefore leading to smaller VDZs widths [25].

The GBs/interfaces character also has an important effect on the formation of VDZs [11,25]. Strong dependence of the VDZs width on GBs characters in irradiated copper has been investigated using TEM [25], since the width of VDZs is an effective reflection of the sink efficiency of GBs. The experimental results show that, in irradiated copper, the VDZs widths generally increase with the increase of the GB plane inclination angle for Σ3 tilt GBs, and with the increase of the misorientation angle for non-Σ3 GBs [25]. Norris [18] reported that, during electron irradiation, an incoherent boundary in nickel is a good vacancy sink and suppresses void growth in its vicinity, and concluded that void growth is enhanced where the boundary is coherent while suppressed where the boundary is incoherent. However, Chen and Buttry [11] reported the appearance of VDZs on both sides of the coherent and incoherent twin boundaries. Many twin bands in ion irradiated nickel were absolutely depleted of voids, since the twin bands were not wide enough to escape the coverage by the VDZs from both interfaces [11]. Moreover, irradiation induced cavities are observed predominantly along GBs in NS copper but less in the grain interior, since the grain size of the NS copper (~50 nm or less after irradiation) is smaller than twice of the characteristic widths of VDZs [28]. Hence, further studies are required to determine the role of interface coherency in the absorption of point defects during irradiation.

However, the existence of VDZs adjacent to the GBs is not an invariable feature [31], since denuded zones are absent in many observations. Lane and Goodhew [32] found that all interfaces except the coherent twins are preferred nucleation sites for bubbles in a ternary austenite steel after helium irradiation in the temperature range 450–600 °C, and the bubble density is greater on interfaces containing resolvable grain boundary dislocations (GBDs). There is no obvious VDZs near GBs in copper after irradiation at 300 °C, and the void size at GBs is slightly larger than that in the grain interiors [25]. In addition, void formation in the GBs was reported recently in neutron-irradiated copper where VDZs were also observed [25], and enrichment of cavities along GBs was also observed in irradiated nickel alloy [27]. Microstructure analysis of helium irradiated T91 martensitic steel [33] also show that voids could form on prior austenite GBs and other interfaces, such as sub-GBs, carbide-matrix interface, lath boundaries, dislocations inside the lath structures, while no preferential nucleation site was observed when the helium implantation was performed at room temperature (RT) [34]. The possible reason for this phenomenon is that helium could diffuse over long distance in the matrix due to the high temperature (550 °C). In addition, a typical void distribution formed near a coincidence site lattice boundary (CSLB) in Fe-15Cr-15Ni steel after neutron irradiation was observed recently, although VDZs were formed near the random GBs [16]. The absence of VDZs near the CSLB would be caused by less vacancy flow towards the CSLB due to the low sink strength of CSLB [16].

It is reasonable to conclude that the appearance of VDZs and the unusual behavior of void formation near GBs occur in metals under certain irradiation conditions, such as irradiation dose, dose rate, temperature and GB characters [11,25]. But the onset of this behavior is relatively insensitive to the energy and species of the particles that are used for irradiation.

## 3. Irradiation Resistance of NS Materials

In the past twenty years, many works have been done to investigate the irradiation resistance of NS materials [3,4,6]. The better radiation stability of NS materials, compared with the corresponding CG materials, was first marked for the nanocrystals of ZrO_2_ and Pd irradiated by Kr ions [35,36], during which the interfaces were regarded as the sinks for the radiation defects. Due to the complex irradiation conditions and various NS materials, however, irradiation leads to many inconsistent changes both in microstructure evolutions and mechanical properties [3,4,37], even though some of the NS materials really have excellent stability under irradiation. According to the general considerations and existing experimental data, the irradiation resistance of NS materials can be proposed as follows [4].

(1) The NS materials have better irradiation stability due to the sinks for the irradiation defects in GBs/interface.

(2) The NS materials transformed to an amorphous state.

(3) Other phenomena, such as irradiation induced alloying component segregation, irradiation induced recrystallization and formation of nanocrystals induced by irradiation in metallic glasses.

**Figure 2:**
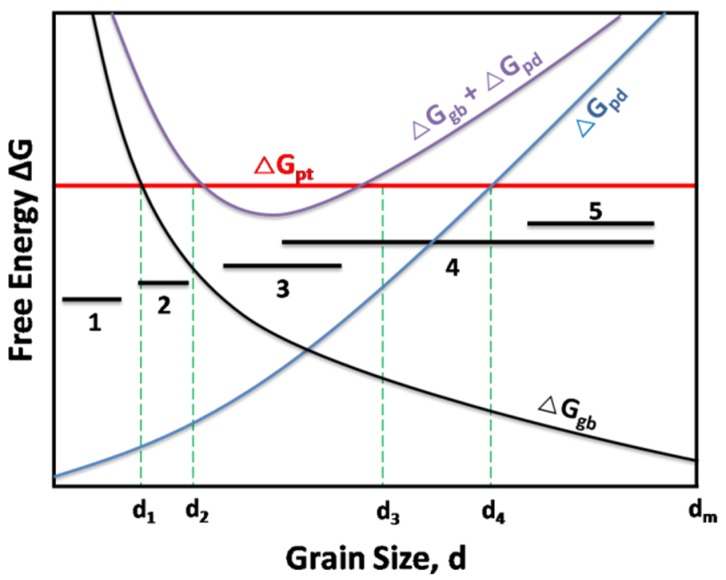
Schematic descriptions for the free energy of point defects (ΔG_pd_), grain boundaries (ΔG_gb_) and phase-transition (ΔG_pt_) in irradiated nanostructured materials as a function of grain size (d) [38].

An energetic approach [38,39], taking into account the sums of the free energy caused by GBs and point defects produced during irradiation, as well as the free energy barrier of amorphization, was developed to illustrate the microstructure evolution of NS materials during irradiation. According to this theory, there is a special optimal grain size for each NS material that provides the most effective amorphization resistance. As schematically shown in Figure 2, there are five zones according to the grain size of NS materials [4,38].

(1) The transformation to the amorphous state occurs without any irradiation (d < d_1_).

(2) A weak irradiation could lead to the transformation to the amorphous state (d_1_ < d < d_2_).

(3) An irradiation does not lead to amorphization (d_2_ < d < d_3_).

(4) An irradiation can lead to amorphization (d_3_ < d < d_4_).

(5) The boundaries play a limited role on the total free energy (d_4_ < d < d_m_), and the defect annihilation by a volume recombination is dominating, which is similar to CG materials (d > d_m_).

The intra-grain defect clusters in NS materials are strongly dependent on the grain size. Experimental observation of NS Ti produced by equal channel angular pressing (ECAP) [40] showed that, subgrains and dislocation cells may be found in large-sized grains (>320 nm), while cell structure without intermediate subgrains may be observed in medium-sized grains (130–600 nm). However, no dislocation cells were observed in grains and subgrains smaller than 150 nm, and grains smaller than 100 nm could be dislocation free in their interiors. In addition, the irradiation-produced point defects can easily move to the GBs/interfaces due to the extremely small grain sizes. Therefore, the grain size and character of the GBs or sub-GBs in NS materials play dominant roles in the microstructure evolution during irradiation [41].

In the following part of this section, much attention will be paid on the enhanced irradiation resistance, irradiation induced amorphization and irradiation induced grain growth in NS materials, since the investigations and understanding of irradiation-induced segregations in NS materials just began [4], and the formation of nanocrystals induced by irradiation in metallic glasses is beyond the scope of the present review.

### 3.1. Enhanced Irradiation Resistance

NS materials with enhanced irradiation resistance have been widely observed in experiments. Nanocrystalline MgGa_2_O_4_ possesses a much higher irradiation tolerance than that in CG microstructures with an average grain size of 10 µm [42]. The nanocrystalline Ti_49.4_Ni_50.6_ alloys retained the long-range order, while amorphization was observed in the large-grained region after being irradiated with 1.5 MeV Ar^+^ ions at RT [43]. Microstructure evolution of ultrafine grained (UFG) and CG Fe-14Cr-16Ni (wt %) alloy subject to helium ion irradiation were investigated, showing that the density of helium bubbles, dislocation loops as well as radiation induced hardening were reduced in the UFG alloy comparing to those in the CG counterpart, which implied that radiation tolerance in UFG alloy is better than that in the corresponding bulk metals [44]. Experimental results of helium ion irradiated nanocrystalline Fe also showed that smaller grains lead to lower density of helium bubbles, and irradiation induced hardening in nanocrystalline Fe is much less than that in bulk Fe [45].

**Figure 3:**
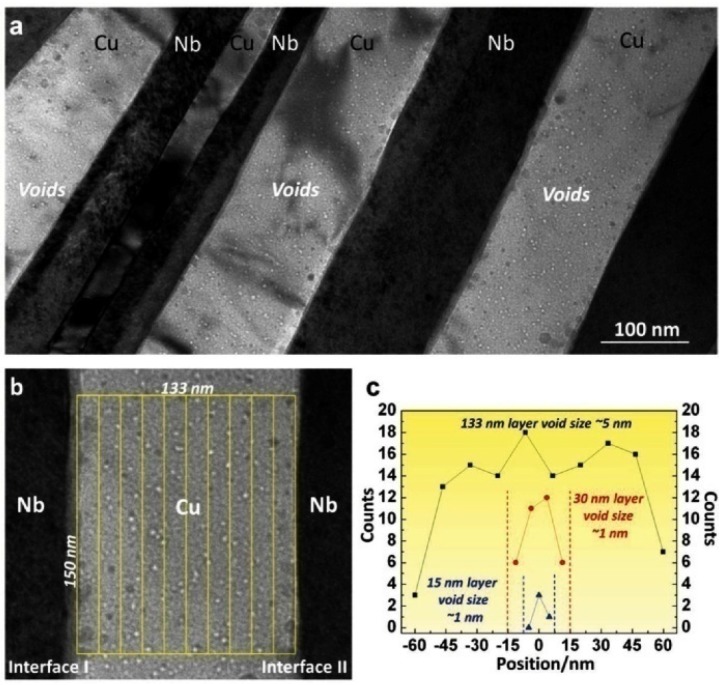
(**a**) Irradiation-induced voids in Cu layers in irradiated accumulative roll bonding (ARB) nanolayered (NL) Cu-Nb composites with individual layer thickness of 135 nm; (**b**) illustration of the method to determine the void number density in Cu layers; and (**c**) plot of the number density of voids as a function of distance from the center of the layer in 133 nm-, 30 nm-, and 15 nm-thick Cu layers [47].

Microstructure evolutions of multilayer NS films (for example, Cu/Nb [46,47,48], Fe/W [49], Cu/V [50,51], Al/Nb [52]) irradiated with helium ions were investigated in the past decades, and NS films also showed excellent irradiation resistance [7]. As shown in Figure 3, morphology of irradiated Cu/Nb multilayer showed that the interface between face-centered-cubic copper and body-centered-cubic Nb can be effective sinks for irradiation induced defects, and VDZs are present near the Cu-Nb interfaces, but the VDZs widths differ from interface to interface due to different vacancy sink efficiency, which is a function of interface crystallographic character [47]. However, helium bubbles are observed both along interfaces and inside the layers in irradiated Cu/V multilayer at a fluence of 6 × 10^16^ cm^−2^ [51]. An obvious damage layer, consisting of high density dislocation clusters and vacancies, was observed in the bottom of helium irradiated TiN film at a dose of 4.0 × 10^16^ cm^−2^, and further studies showed that TiN films with smaller grains have better radiation resistance than that of the materials with larger grains [53]. However, for all multilayer films studied, irrespective of the interface character, the concentration of helium nanoinclusions with size of 1–2 nm decreases with decreasing layer thickness [3].

Computational and experimental studies showed that the strength of interfacial sinks for irradiation-produced point defects can depend strongly on the interface character, such as the crystallography and chemistry [25,28,54,55,56]. The width of VDZs around GBs and Cu-Nb interfaces of different crystallographic character showed that high-angle GBs are good point defect sinks, while coherent twin boundaries, low-angle GBs, as well as Kurdjumov–Sachs (KS) interfaces are poorer sinks for the irradiation-produced point defects [25,56]. Studies of irradiation creep on NS Cu samples also showed that high-energy GBs are more effective sink sites than the KS interfaces between Cu and W nanoprecipitates [57,58]. Recent experimental results showed that the average point defect absorption probability of Cu-Nb KS interfaces should be the highest, while that of Cu-Ni interfaces were the lowest, and the values for Cu-V KS interfaces are intermediate [56].

The enhanced irradiation resistance of NS materials can be attributed to the large surface area of GBs/interfaces, which can act as sinks for the annihilation of point defects, such as interstitials and vacancies created during irradiation. Sun *et al.* [59] provided direct evidence, via *in situ* Kr ions irradiation within a TEM, that high angle GBs in NS nickel, with grain size of ~55 nm, could effectively absorb the dislocation loops and segments produced during irradiation. Their study also showed that the high angle GBs can significantly reduce the size and density of irradiation induced defect clusters in NS Ni compared to the corresponding bulk counterparts, indicating that NS Ni has significant enhancement of irradiation tolerance. Molecular dynamics (MD) simulation showed that the GBs have a surprising “loading-unloading” effect during irradiation process [60]. The irradiation induced interstitials pass into the boundaries at first, which act as a source, then the boundaries emit interstitials into the inner of the grain to annihilate vacancies. The “loading-unloading” mechanism of interstitial emission during irradiation may help to explain the phenomenon that NS materials have better irradiation resistance than bulk materials, since the NS materials have more GBs than that in bulk materials.

### 3.2. Irradiation Induced Amorphization

It is known that ceramic materials, especially those with complex structures and compositions, are susceptible to irradiation induced amorphization [61,62]. Wang *et al.* [63,64] investigated ion irradiation-induced amorphization in ceramic materials with complex structure using *in situ* TEM observation. Their experimental results showed that there is a critical amorphization temperature, above which amorphization would not occur in ceramics of garnet structure types [63], and critical amorphization dose increases with increasing irradiation temperature at a rate determined by the kinetics of the amporphization and crystallization process [64]. In addition, complete amorphization of SiC, which was neutron irradiated at ~70 °C to a dose of ~2.56 dpa, was also observed using high resolution transmission electron microscopy (HRTEM) [65].

**Figure 4:**
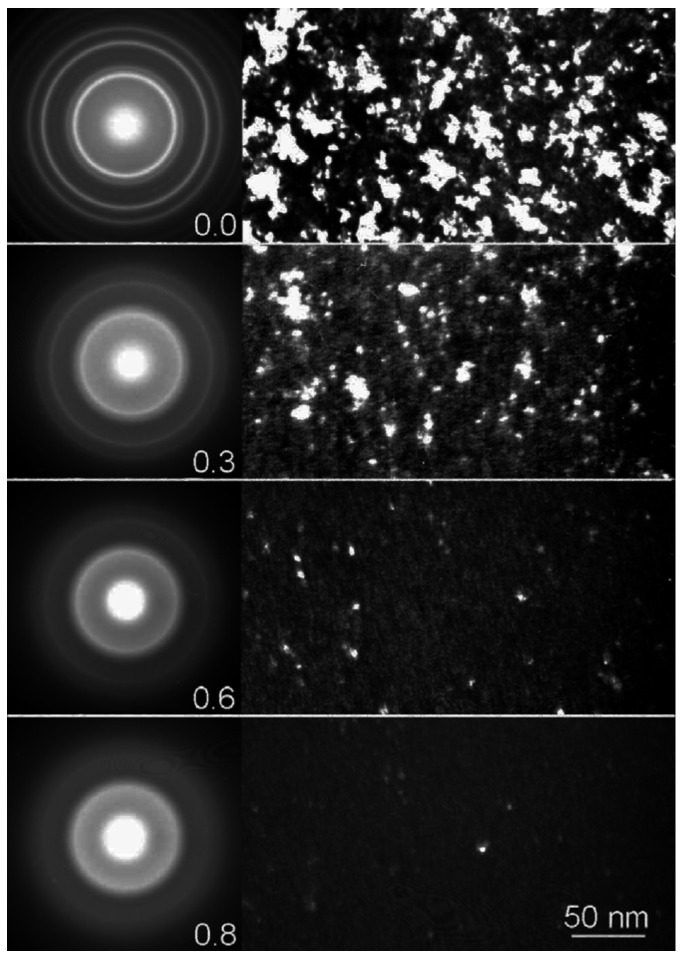
Sequence of dark-field TEM images and associated electron-diffraction patterns showing the effects of Xe-ion irradiation on nanocrystalline ZrO_2_. The number in the bottom right corner of each diffraction pattern is the ion dose in dpa. The dark-field images were taken with the objective aperture centered over the bright (111) diffraction ring [67].

There are also some adverse reports that some NS materials show a negative effect on irradiation tolerance, such as irradiation-induced amorphization, compared to their bulk counterparts. For instance, nanocrystalline zirconia (ZrO_2_) nanoparticles can be amorphized at a dose as low as 0.9 dpa, while no amorphization was observed in bulk zirconia irradiated to 680 dpa [66]. Meldrum *et al.* [67] studied the irradiation effects of nanocrystalllie ZrO_2_ particles embedded in a surrounding matrix of amorphous SiO_2_. As shown in Figure 4, the bright area in the TEM dark-field images, corresponding to the crystalline region, began to disappear at a dose of 0.3 dpa; while the electron-diffraction halo, corresponding to the amorphous materials, become clearer. With the increase of dpa, the rings, corresponding to the tetragonal ZrO_2_ phase completely disappeared, and the polycrystalline rings specific to the cubic phase became fainter gradually. The nanocomposite ZrO_2_ becomes completely amorphous at a dose of 0.9 dpa. However, no evidence of amorphization was observed on bulk ZrO_2_ that was irradiated to doses as high as 110 dpa [68]. NS polycrystalline 3C-SiC films with 3.8 nm in average grain size were also amorphous when the samples were irradiated by 1 MeV Si^+^ at RT [69]. MD simulation of electron-irradiation of NiZr_2_ showed that both random atom exchanges and Frenkel-pair introduction can lead to the amorphization [70]. Although some models of irradiation-induced amorphization in ceramics have been established [71], both systematic experimental studies and theoretical analysis of irradiation-induced amorphization in NS materials are still needed in the future.

### 3.3. Irradiation Induced Grain Growth

Irradiation induced grain growth has also been extensively investigated in the past decades. As illustrated by NS copper and nickel produced by severe plastic deformation (SPD), an irradiation induced grain growth in NS Cu–0.5Al_2_O_3_ from 178 to 493 nm was observed [72,73]. In addition, the increase of average grain size from 40 nm to 60 nm was observed in the NS austenite steel SW316SS after irradiated with Fe ions [74]. Irradiation induced grain growth was also reported in NS ferrite/martensite steel produced by surface mechanical attrition treatment (SMAT) [75]. Moreover, irradiation induced grain growth has also been widely observed in nanocrystalline thin films (for example, Zr NS film [76], Fe/Fe (Zr) NS films [77], Ni film [78], Pd film [79], Zr-Fe NS film [80] and Zr, Pt, Cu, and Au NS films [81]).

**Figure 5:**
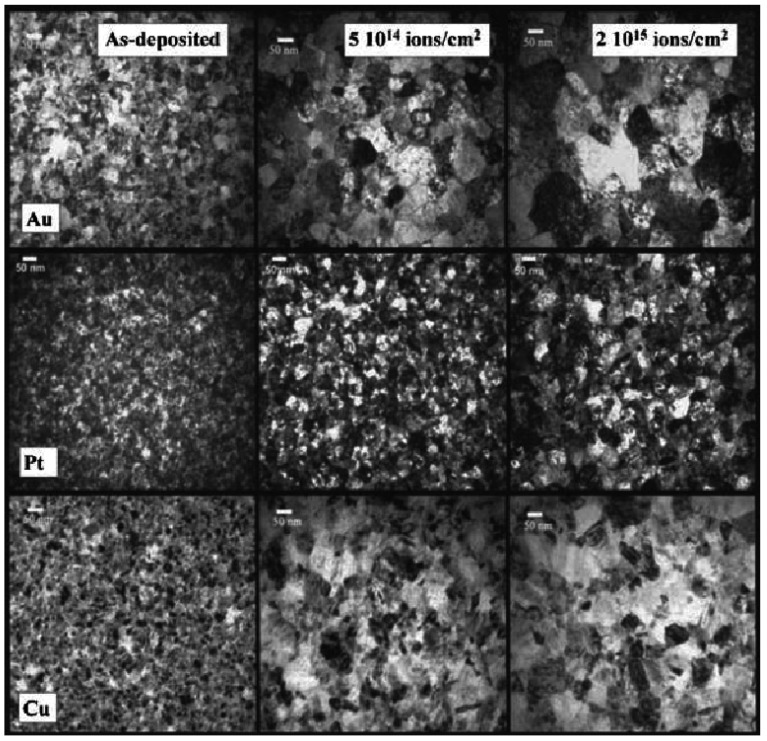
Sequence of bright-field TEM images taken at different ion doses showing grain-growth induced by ion irradiation at room temperature; from top to bottom: pure Au thin-film irradiated with 500 keV Ar ions, Ptthin-film irradiated with 500 keV Ar ions, and Cu thin-film irradiated with 500 keV Kr ions [81]. The scale mark is 50 nm.

Temperature has an important effect on the irradiation induced grain growth behavior [6,80,81]. Irradiation induced grain growth in NS metallic films (Zr, Pt, Cu, and Au) was investigated in detail using Kr (E = 0.5–1 MeV) and Ar (E = 0.5 MeV) ions irradiation, and grain sizes were found to increase at all studied temperatures between 20 and 573 K, including the low-temperature regime (<0.15 − 0.22*T*_m_, *T*_m_ is the melting point temperature), where grain growth is independent of the irradiation temperature [81]. As shown in Figure 5, grain-growth induced by ion irradiation at room temperature was observed using bright-field TEM imaging [81]. Based on these experimental results, the temperature ranges, where the irradiation induced grain growth occurs, can be divided into three intervals: low temperature region (where the irradiation action is dominating and the temperature influence is insignificant), thermally assisted regime (corresponding to a combination of the irradiation and thermal action) and pure thermal region (where the temperature influence in the recrystallization is dominating). The grain growth behavior seems to be independent of temperature in the low temperature region. However, the rate of grain growth increases with increasing temperature in the other two regions. The thermal spike model, based on the direct impact of the thermal spikes on GBs, can be used to account for the grain growth behavior under irradiation in the low temperature region [81,82]. According to this theory, the thermal peaks are formed in the cascades and sub-cascades during irradiation, which causes the migration of GBs and thus leads to the grain growth under irradiation. MD simulation results showed that irradiation induced grain growth occurs if the volume of the thermal spike is larger than the grain volume or overlaps the GB area; while no grain growth was observed if the volume of thermal spike does not reach the GB area [83].

**Figure 6:**
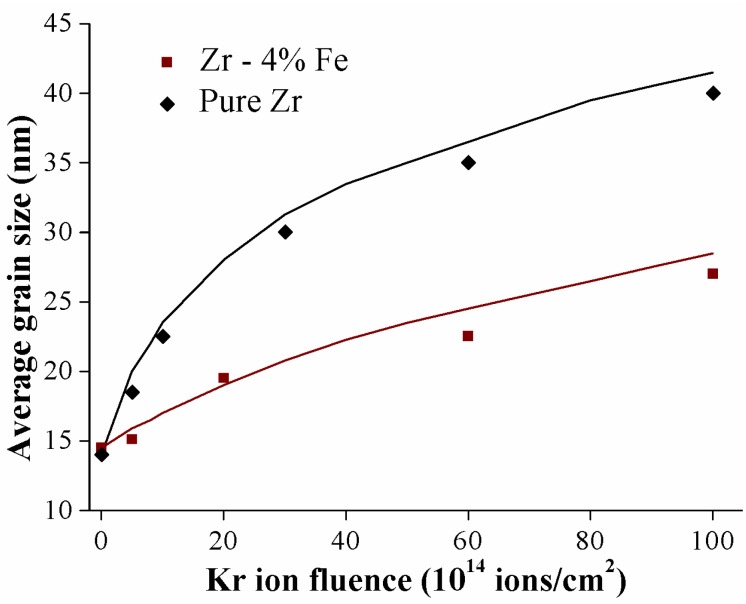
Average grain size *versus* ion fluence for pure Zr and Zr-1.2%Fe irradiated with 500 keV Kr ions at 20 K [80].

It is reported that there is also an effect of irradiation flux and dose on the changes of grain size of NS materials under irradiation. As shown in Figure 6, significant grain growth was observed in Zr-Fe nanocrystalline thin films after irradiation with 500 keV Kr ions at irradiation temperatures of 20 K, with fluence in excess of 10^16^ ions/cm^2^ [80]. According to the experimental results, the average grain size after irradiation increases monotonically with ion fluence until it reaches a saturation value, which depends both on the property of the films and on temperature. However, the grain growth rate of Cu thin film is independent of ion flux, and it is suggested that this ion irradiation induced grain growth is associated with the thermal spike diffusion [82]. In addition, the average grain size *L* in Ar^+^ irradiated Cu films at RT increases with increasing ion dose, following a relationship of *L*^n^–*L*_0_^n^ ~*K*(*ϕ*) type, where *L*_0_ is the initial grain size, *t* is time, *ϕ* is ion dose, and *K* is a constant, depending on the GB mobility and driving force of grain growth, *n*~3.3 [82].

Both experimental observations and kinetic Monte Carlo (KMC) simulations showed that the irradiation-induced nanoprecipitation in NS materials can significantly affect the microstructure stability of NS materials during irradiation and post-irradiation annealing (for example, Cu-Nb-W alloys [84,85], Ni-W alloys [86] and Cu-Ag-W alloys [87]). W precipitates formed during room temperature irradiation in Cu-Nb-W alloy are extremely resistant to coarsening during the post-irradiation annealing [85]. The W precipitates, formed during room temperature irradiation in Cu_83.5_Ag_15_W_1.5_ alloy, serve as irradiation-induced point defect sinks which lead to an extension of the compositional patterning regime in moderate-temperature regime (≤300 °C); while they serve as pinning sites to stop the growth of Ag nanoprecipitates in high-temperature regime (≥400 °C), and both mechanisms are active between 300 and 400 °C [87].

It must be pointed out that in some studies the grain sizes decrease under irradiation. The decrease of grain sizes during irradiation from 115 nm to 38 nm was observed in protons and nickel ions irradiated NS nickel sample produced by SPD [72,73]. It is suggested that the defect clusters produced during irradiation move to the GBs and form a cell structure, which may result in the formation of new smaller grains [72]. The decrease of grain sizes from 35–40 nm to 15–20 nm in bismuth doped BaS was also observed after 120 MeV Ni ion irradiation [88]. Similar results were also observed in TiN/Si bilayers after irradiated with 200 keV Ar^+^ at RT [89]. Moreover, grain growth would not happen in Zr films even after high dose irradiation (180 dpa) with high energy electrons [81].

It is reasonable to conclude that the changes of grain size in the irradiated NS materials are determined both by the irradiation condition, such as irradiation dose, flux and temperature, and by the properties of the NS materials, such as initial grain size and other factors. However, more efforts are still on demand to further clarify these processes.

## 4. Summary and Critical Assessment

The GBs/interfaces, which can be effective sinks for the irradiation induced point defects, play an important role in the irradiation process of materials. Formation of VDZs near GBs/interfaces, due to the sinks of irradiation-produced point defects, was widely observed in various NS materials and NS multilayer materials. The appearance of VDZs and the unusual behavior of void formation near GBs in metals under certain irradiation conditions, such as irradiation dose, dose rate, temperature and GBs character. However, it is not an invariable feature for the existence of VDZs adjacent to the GBs, due to the different properties of NS materials and different irradiation conditions.

Irradiation resistance of NS materials has been extensively investigated. Some experimental results show that NS materials exhibit excellent irradiation resistance compared with their bulk counterparts, while some experimental results show that NS materials are easily amorphized during irradiation due to the crystal lattice instability. On the one hand, GBs/interfaces act as effective sinks of irradiation-produced point defects, in which case the NS materials may have enhanced irradiation tolerance. On the other hand, GBs/interfaces are characterized by excess energy (compared with that of the GBs-free crystals), in which case the GBs/interfaces provide a shift in the total free energy and hence enhances irradiation-induced amorphization. It seems that there exists an optimal grain size, where the materials have the best irradiation tolerance, for each specific NS materials. To gain a complete understanding of the irradiation effects on NS materials, however, both systematic experimental studies and computer simulations should be carried out to establish the role of the size effects and GBs/interfaces on the irradiation resistance in NS materials, since the effect of grain size and GBs/interfaces on the irradiated NS materials could be non-monotonic.

## Abbreviations

The following abbreviations are used in this manuscript:

ARB: accumulative roll bonding

CG: coarse-grained

CSLB: coincidence site lattice boundary

ECAP: equal channel angular pressing

GBs: grain boundaries

HRTEM: high resolution transmission electron microscopy

IAV: interstitial atoms and vacancies

KMC: kinetic Monte Carlo

KS: Kurdjumov–Sachs

MD: molecular dynamics

NL: nanolayered

NS: nanostructured

RT: room temperature

SMAT: surface mechanical attrition treatment

SPD: severe plastic deformation

TEM: transmission electron microscopy

VDZs: void denuded zones

## References

[1] Gleiter H. (2000). Nanostructured materials: Basic Concepts and Microstructure. Acta Mater..

[2] Baretzky B., Baro M., Grabovetskaya G., Gubicza J., Ivanov M., Kolobov Y.R., Langdon T., Lendvai J., Lipnitskii A., Mazilkin A. (2005). Fundamentals of interface phenomena in advanced bulk nanoscale materials. Rev. Adv. Mater. Sci..

[3] Andrievskii R. (2010). Effect of irradiation on the properties of nanomaterials. Phys. Met. Metallogr..

[4] Andrievski R. (2011). Behavior of radiation defects in nanomaterials. Rev. Adv. Mater. Sci..

[5] Was G.S. (2007). Fundamentals of Radiation Materials Science.

[6] Chang Y.-Q., Guo Q., Zhang J., Chen L., Long Y., Wan F.-R. (2013). Irradiation effects on nanocrystalline materials. Front. Mater. Sci..

[7] Misra A., Demkowicz M., Zhang X., Hoagland R. (2007). The radiation damage tolerance of ultra-high strength nanolayered composites. JOM.

[8] Mastel B., Brimhall J. (1968). Voids produced in high purity nickel by neutron irradiation. J. Nucl. Mater..

[9] Norris D. (1972). Voids in irradiated metals (part I). Radiat. Eff..

[10] Norris D. (1970). Voids in nickel irradiated with electrons after previous argon ion bombardment. Science.

[11] Chen C., Buttry R. (1981). Void formation and denudation in ion-irradiated nickel. Radiat. Eff..

[12] Shaikh M. (1992). Void denudation and grain boundary migration in ion-irradiated nickel. J. Nucl. Mater..

[13] Singh B., Horsewell A., Gelles D., Garner F. (1992). Void swelling in copper and copper alloys irradiated with fission neutrons. J. Nucl. Mater..

[14] Zinkle S.J., Farrell K. (1989). Void swelling and defect cluster formation in reactor-irradiated copper. J. Nucl. Mater..

[15] Kim I.-S., Hunn J., Hashimoto N., Larson D., Maziasz P., Miyahara K., Lee E. (2000). Defect and void evolution in oxide dispersion strengthened ferritic steels under 3.2 MeV Fe+ ion irradiation with simultaneous helium injection. J. Nucl. Mater..

[16] Sekio Y., Yamashita S., Sakaguchi N., Takahashi H. (2015). Void denuded zone formation for Fe-15Cr-15Ni steel and PNC316 stainless steel under neutron and electron irradiations. J. Nucl. Mater..

[17] Shimada M., Kamei H. (1981). Ion irradiation mode effects on void formation. J. Nucl. Mater..

[18] Norris D. (1971). The use of the high voltage electron microscope to simulate fast neutron-induced void swelling in metals. J. Nucl. Mater..

[19] Brimhall J., Mastel B. (1969). Stability of voids in neutron irradiated nickel. J. Nucl. Mater..

[20] Alexander D.E., Rehn L., Baldo P., Gao Y. (1993). Enhancement of diffusion-induced grain boundary migration by ion irradiation. Appl. Phys. Lett..

[21] Watanabe S., Sakaguchi N., Hashimoto N., Nakamura M., Takahashi H., Namba C., Lam N. (1996). Radiation-induced segregation accompanied by grain boundary migration in austenitic stainless steel. J. Nucl. Mater..

[22] Watanabe S., Sakaguchi N., Hashimoto N., Takahashi H. (1995). Quantitative studies of irradiation-induced segregation and grain boundary migration in FeCrNi alloy. J. Nucl. Mater..

[23] Greenwood G., Foreman A., Rimmer D. (1959). The role of vacancies and dislocations in the nucleation and growth of gas bubbles in irradiated fissile material. J. Nucl. Mater..

[24] Norris D. (1971). The growth of voids in nickel in a high—Voltage electron microscope. Philos. Mag..

[25] Han W., Demkowicz M., Fu E., Wang Y., Misra A. (2012). Effect of grain boundary character on sink efficiency. Acta Mater..

[26] Zhang H., Yao Z., Daymond M.R., Kirk M.A. (2014). Cavity morphology in a Ni based superalloy under heavy ion irradiation with cold pre-injected helium. I. J. Appl. Phys..

[27] Zhang H., Yao Z., Daymond M.R., Kirk M.A. (2014). Cavity morphology in a Ni based superalloy under heavy ion irradiation with hot pre-injected helium. Ii. J. Appl. Phys..

[28] Han W., Fu E., Demkowicz M.J., Wang Y., Misra A. (2013). Irradiation damage of single crystal, coarse-grained, and nanograined copper under helium bombardment at 450 °C. J. Mater. Res..

[29] Zinkle S. (1994). Microstructure of ion irradiated ceramic insulators. Nucl. Instrum. Methods Phys. Res. Sect. B.

[30] Stiegler J., Mansur L. (1979). Radiation effects in structural materials. Annu. Rev. Mater. Sci..

[31] Farrell K., Houston J., Wolfenden A., King R., Jostsons A. (1971). Effects of Structural Imperfections on Voids in Aluminum.

[32] Lane P., Goodhew P. (1983). Helium bubble nucleation at grain boundaries. Philos. Mag. A.

[33] Henry J., Mathon M.-H., Jung P. (2003). Microstructural analysis of 9% Cr martensitic steels containing 0.5 at.% helium. J. Nucl. Mater..

[34] Jiao Z., Ham N., Was G. (2007). Microstructure of helium-implanted and proton-irradiated T91ferritic/martensitic steel. J. Nucl. Mater..

[35] Rose M., Balogh A., Hahn H. (1997). Instability of irradiation induced defects in nanostructured materials. Nucl. Instrum. Methods Phys. Res. Sect. B.

[36] Rose M., Gorzawski G., Miehe G., Balogh A., Hahn H. (1995). Phase stability of nanostructured materials under heavy ion irradiation. Nanostruct. Mater..

[37] Ovid’Ko I., Sheinerman A. (2005). Irradiation-induced amorphization processes in nanocrystalline solids. Appl. Phys. A.

[38] Shen T. (2008). Radiation tolerance in a nanostructure: Is Smaller Better?. Nucl. Instrum. Methods Phys. Res. Sect. B.

[39] Wurster S., Pippan R. (2009). Nanostructured metals under irradiation. Scr. Mater..

[40] Zhu Y., Huang J., Gubicza J., Ungár T., Wang Y., Ma E., Valiev R. (2003). Nanostructures in Ti processed by severe plastic deformation. J. Mater. Res..

[41] Samaras M., Derlet P., Van Swygenhoven H., Victoria M. (2002). Computer simulation of displacement cascades in nanocrystalline Ni. Phys. Rev. Lett..

[42] Shen T.D., Shihai F., Ming T., Valdez J.A., Yongqiang W., Sickafus K.E. (2007). Enhanced radiation tolerance in nanocrystalline MgGa_2_O_4_. Appl. Phys. Lett..

[43] Kilmametov A., Gunderov D., Valiev R., Balogh A., Hahn H. (2008). Enhanced ion irradiation resistance of bulk nanocrystallineTiNi alloy. Scr. Mater..

[44] Sun C., Yu K., Lee J., Liu Y., Wang H., Shao L., Maloy S., Hartwig K., Zhang X. (2012). Enhanced radiation tolerance of ultrafine grained Fe-Cr-Ni alloy. J. Nucl. Mater..

[45] Yu K., Liu Y., Sun C., Wang H., Shao L., Fu E., Zhang X. (2012). Radiation damage in helium ion irradiated nanocrystalline Fe. J. Nucl. Mater..

[46] Zhernenkov M., Jablin M.S., Misra A., Nastasi M., Wang Y., Demkowicz M.J., Baldwin J.K., Majewski J. (2011). Trapping of implanted he at Cu/Nb interfaces measured by neutron reflectometry. Appl. Phys. Lett..

[47] Han W., Demkowicz M.J., Mara N.A., Fu E., Sinha S., Rollett A.D., Wang Y., Carpenter J.S., Beyerlein I.J., Misra A. (2013). Design of radiation tolerant materials via interface engineering. Adv. Mater..

[48] Lach T.G., Ekiz E.H., Averback R.S., Mara N.A., Bellon P. (2015). Role of interfaces on the trapping of he in 2D and 3DCu-Nbnanocomposites. J. Nucl. Mater..

[49] Li N., Fu E., Wang H., Carter J., Shao L., Maloy S., Misra A., Zhang X. (2009). He ion irradiation damage in Fe/Wnanolayer films. J. Nucl. Mater..

[50] Fu E., Carter J., Swadener G., Misra A., Shao L., Wang H., Zhang X. (2009). Size dependent enhancement of helium ion irradiation tolerance in sputtered Cu/V nanolaminates. J. Nucl. Mater..

[51] Zhang X., Fu E., Misra A., Demkowicz M. (2010). Interface-enabled defect reduction in He ion irradiated metallic multilayers. JOM.

[52] Li N., Martin M., Anderoglu O., Misra A., Shao L., Wang H., Zhang X. (2009). He ion irradiation damage in Al/Nb multilayers. J. Appl. Phys..

[53] Wang H., Araujo R., Swadener J., Wang Y., Zhang X., Fu E., Cagin T. (2007). Ion irradiation effects in nanocrystalline tin coatings. Nucl. Instrum. Methods Phys. Res. Sect. B.

[54] Demkowicz M., Hoagland R., Hirth J. (2008). Interface structure and radiation damage resistance in Cu-Nb multilayer nanocomposites. Phys. Rev. Lett..

[55] Mao S., Dillon S., Averback R.S. (2013). The influence of Cu-Nb interfaces on local vacancy concentrations in Cu. Scr. Mater..

[56] Mao S., Shu S., Zhou J., Averback R.S., Dillon S.J. (2015). Quantitative comparison of sink efficiency of Cu-Nb, Cu-V and Cu-Ni interfaces for point defects. Acta Mater..

[57] Tai K., Averback R.S., Bellon P., Ashkenazy Y. (2011). Irradiation-induced creep in nanostructured Cu alloys. Scripta. Mater..

[58] Tai K., Averback R.S., Bellon P., Ashkenazy Y., Stumphy B. (2012). Temperature dependence of irradiation-induced creep in dilute nanostructured Cu-W alloys. J. Nucl. Mater..

[59] Sun C., Song M., Yu K., Chen Y., Kirk M., Li M., Wang H., Zhang X. (2013). In situ evidence of defect cluster absorption by grain boundaries in Kr ion irradiated nanocrystallineNi. Metall. Mater. Trans. A.

[60] Bai X.-M., Voter A.F., Hoagland R.G., Nastasi M., Uberuaga B.P. (2010). Efficient annealing of radiation damage near grain boundaries via interstitial emission. Science.

[61] Hobbs L.W., Clinard F.W., Zinkle S.J., Ewing R.C. (1994). Radiation effects in ceramics. J. Nucl. Mater..

[62] Matzke H. (1982). Radiation damage in crystalline insulators, oxides and ceramic nuclear fuels. Radiat. Eff..

[63] Utsunomiya S., Wang L., Yudintsev S., Ewing R. (2002). Ion irradiation-induced amorphization and nano-crystal formation in garnets. J. Nucl. Mater..

[64] Wang L., Wang S., Gong W., Ewing R., Weber W. (1998). Amorphization of ceramic materials by ion beam irradiation. Mater. Sci. Eng. A.

[65] Snead L., Zinkle S., Hay J., Osborne M. (1998). Amorphization of sic under ion and neutron irradiation. Nucl. Instrum. Methods Phys. Res. Sect. B.

[66] Meldrum A., Boatner L.A., Ewing R.C. (2003). Size effects in the irradiation-induced crystalline-to-amorphous transformation. Nucl. Instrum. Methods Phys. Res. Sect. B.

[67] Meldrum A., Boatner L., Ewing R. (2001). Nanocrystalline zirconia can be amorphized by ion irradiation. Phys. Rev. Lett..

[68] Fleischer E.L., Norton M.G., Zaleski M.A., Hertl W., Carter C.B., Mayer J.W. (1991). Microstructure of hardened and softened zirconia after xenon implantation. J. Mater. Res..

[69] Jiang W., Wang H., Kim I., Zhang Y., Weber W.J. (2010). Amorphization of nanocrystalline 3C-SiC irradiated with Si+ ions. J. Mater. Res..

[70] Devanathan R., Lam N., Okamoto P., Meshii M. (1993). Molecular-dynamics simulation of electron-irradiation-induced amorphization of NiZr_2_. Phys. Rev. B.

[71] Weber W. (2000). Models and mechanisms of irradiation-induced amorphization in ceramics. Nucl. Instrum. Methods Phys. Res. Sect. B.

[72] Nita N., Schaeublin R., Victoria M. (2004). Impact of irradiation on the microstructure of nanocrystalline materials. J. Nucl. Mater..

[73] Nita N., Schaeublin R., Victoria M., Valiev R. (2005). Effects of irradiation on the microstructure and mechanical properties of nanostructured materials. Philos. Mag..

[74] Radiguet B., Etienne A., Pareige P., Sauvage X., Valiev R. (2008). Irradiation behavior of nanostructured 316 austenitic stainless steel. J. Mater. Sci..

[75] Liu W., Zhang C., Ji Y., Yang Z., Zang H., Shen T., Chen L. (2014). Irradiation-induced grain growth in nanocrystalline reduced activation ferrite/martensite steel. Appl. Phys. Lett..

[76] Zhang Y., Jiang W., Wang C., Namavar F., Edmondson P.D., Zhu Z., Gao F., Lian J., Weber W.J. (2010). Grain growth and phase stability of nanocrystalline cubic zirconia under ion irradiation. Phys. Rev. B.

[77] Karpe N., Bøttiger J., Chechenin N., Krog J. (1994). Ion irradiation induced grain growth in nanocrystallineFe and Fe (Zr). Mater. Sci. Eng. A.

[78] Liu J.C., Mayer J. (1987). Ion irradiation induced grain growth in Ni polycrystalline thin films. Nucl. Instrum. Methods Phys. Res. Sect. B.

[79] Liu J.C., Nastasi M., Mayer J. (1987). Ion irradiation induced grain growth in Pd polycrystalline thin films. J. Appl. Phys..

[80] Kaoumi D., Motta A., Birtcher R. (2006). Grain growth in Zr-Fe thin films during *in situ* ion irradiation in a TEM. Nucl. Instrum. Methods Phys. Res. Sect. B.

[81] Kaoumi D., Motta A., Birtcher R. (2008). A thermal spike model of grain growth under irradiation. J. Appl. Phys..

[82] Liu J.C., Li J., Mayer J. (1990). Temperature effect on ion—irradiation—induced grain growth in Cu thin films. J. Appl. Phys..

[83] Voegeli W., Albe K., Hahn H. (2003). Simulation of grain growth in nanocrystalline nickel induced by ion irradiation. Nucl. Instrum. Methods Phys. Res. Sect. B.

[84] Zhang X., Vo N., Bellon P., Averback R. (2011). Microstructural stability of nanostructured Cu-Nb-W alloys during high-temperature annealing and irradiation. Acta Mater..

[85] Zhang X., Wen J., Bellon P., Averback R.S. (2013). Irradiation-induced selective precipitation in Cu-Nb-W alloys: An Approach towards Coarsening Resistance. Acta Mater..

[86] Lee J., Lear C.R., Zhang X., Bellon P., Averback R.S. (2015). Irradiation-induced nanoprecipitation in Ni-W alloys. Metall. Mater. Trans. A.

[87] Zhang X., Shu S., Bellon P., Averback R.S. (2015). Precipitate stability in Cu-Ag–W system under high-temperature irradiation. Acta Mater..

[88] Popović M., Novaković M., Šiljegović M., Bibić N. (2012). Effects of 200kev argon ions irradiation on microstructural properties of titanium nitride films. Nucl. Instr. Methods Phys. Res. Sect. B.

[89] Singh S., Kumar R., Singh N. (2011). Effect of swift heavy ion irradiation on bismuth doped bas nanostructures. J. Alloy. Compd..

